# Distribution of the type III DNA methyltransferases *modA, modB* and *modD* among *Neisseria meningitidis* genotypes: implications for gene regulation and virulence

**DOI:** 10.1038/srep21015

**Published:** 2016-02-12

**Authors:** Aimee Tan, Dorothea M. C. Hill, Odile B. Harrison, Yogitha N. Srikhanta, Michael P. Jennings, Martin C. J. Maiden, Kate L. Seib

**Affiliations:** 1 Institute for Glycomics, Griffith University, Gold Coast, Queensland 4222, Australia; 2 Department of Zoology, University of Oxford, Oxford, UK

## Abstract

*Neisseria meningitidis* is a human-specific bacterium that varies in invasive potential. All meningococci are carried in the nasopharynx, and most genotypes are very infrequently associated with invasive meningococcal disease; however, those belonging to the ‘hyperinvasive lineages’ are more frequently associated with sepsis or meningitis. Genome content is highly conserved between carriage and disease isolates, and differential gene expression has been proposed as a major determinant of the hyperinvasive phenotype. Three phase variable DNA methyltransferases (ModA, ModB and ModD), which mediate epigenetic regulation of distinct *phase vari*able regul*ons* (phasevarions), have been identified in *N. meningitidis*. Each *mod* gene has distinct alleles, defined by their Mod DNA recognition domain, and these target and methylate different DNA sequences, thereby regulating distinct gene sets. Here 211 meningococcal carriage and >1,400 disease isolates were surveyed for the distribution of meningococcal *mod* alleles. While *modA11*-*12* and *modB1-2* were found in most isolates, rarer alleles (*e.g., modA15, modB4, modD1-6)* were specific to particular genotypes as defined by clonal complex. This suggests that phase variable Mod proteins may be associated with distinct phenotypes and hence invasive potential of *N. meningitidis* strains.

*Neisseria meningitidis*, the meningococcus, is a human-specific bacterium that exists as a commensal in approximately 10% of the population^1^; however, some meningococci are associated with severe pathology with rapid disease onset of sepsis and/or meningitis^2^,^3^. Although certain host factors have been identified that contribute to disease susceptibility (including age, medical conditions and genetic factors^2^,^4^,^5^), the precise mechanisms that determine invasive potential and that mediate transition of a given meningococcus from carriage to invasive disease remain unclear. Invasive isolates are typically characterized as one of six capsule-based meningococcal serogroups (A, B, C, X, Y and W). These are globally distributed with varying rates of disease incidence^6^. Meningococci are characterized at the genetic level by multi locus sequence typing (MLST), which uses sequences of seven housekeeping genes to determine isolate sequence type (ST)^7^. Groups of closely related STs are termed clonal complexes (cc) and these are good surrogates of bacterial lineage. A subset of clonal complexes, known as hyperinvasive (or hypervirulent) lineages, is responsible for the majority of disease worldwide. These hyperinvasive lineages are significantly overrepresented in collections of invasive isolates (*i.e.* those from blood or cerebrospinal fluid) relative to collections of asymptomatic carriage isolates (*i.e.,* those from the nasopharynx), and include cc4/5, cc8, cc11, cc32, cc41/44, and cc269^7^,^8^,^9^,^10^. On the other hand, meningococci isolated from asymptomatic carriers consist of highly diverse genotypes with some clonal complexes, such as cc23, cc35, cc106 and cc116, very rarely if ever associated with invasion^10^. Even amongst the hyperinvasive lineages, different genotypes often vary in pathogenicity; for example within the cc41/44 lineage, ST-41 meningococci are more commonly associated with disease, and ST-44 with carriage^10^.

Attempts to identify the specific factors responsible for differential virulence of *N. meningitidis* strains have been largely unsuccessful. Whilst numerous factors important for virulence have been identified (*e.g.* capsule, pili, lipooligosaccharide (LOS) and opacity proteins)^11^, there are no virulence factors that clearly distinguish highly pathogenic isolates that cause invasive disease from less pathogenic isolates. Many proteins considered to be key virulence determinants are also present in *N. meningitidis* carriage isolates and commensal *Neisseria* species^12^,^13^,^14^. Indeed, most meningococcal isolates have a similar overall genome composition, consisting of 79% core genes, 21% accessory genes and less than 0.1% strain specific genes^15^, with a core meningococcal genome of approximately 1600 genes^15^,^16^. Restriction-modification systems are among the few isolate and clonal complex-specific genes identified^15^,^17^. Other genetic elements associated with invasive isolates include the *hmbR* hemoglobin receptor gene^18^ and a prophage^12^,^19^,^20^; however, the mechanistic contribution of these factors to virulence is unclear. Consequently, the pathogenic potential of isolates is hypothesized to be a polygenetic phenomenon arising from varied adhesin and metabolic gene content, and expression differences^21^,^22^.

While meningococci are considered to have relatively few transcriptional regulators when compared to other bacterial species^23^, they do contain numerous phase variable genes^24^,^25^,^26^ and differential gene expression provides a possible explanation for phenotypic differences. Furthermore, *N. meningitidis* contain a number of phase variable DNA methyltransferases (Mod), associated with type III restriction-modification systems, which mediate epigenetic regulation^27^,^28^,^29^,^30^. Random, reversible, hypermutation of repetitive DNA tracts within the open reading frame of *mod* genes lead to frame-shift mutations and the ON/OFF switching of Mod expression (*i.e.,* phase variation). Mod phase variation results in distinct bacterial populations with different patterns of genome methylation, and altered expression of specific sets of genes. These *phase vari*able regul*ons* have been defined as phasevarions^29^,^31^. Mod phasevarions studied to date in pathogenic *Neisseria* have been shown to contain genes encoding outer membrane proteins, stress response proteins and other metabolic components^27^,^28^. These phasevarions represent an epigenetic mechanism by which meningococcal cells can alter complex phenotypes, which may affect carriage or invasion.

Three *mod* genes have been described in *N. meningitidis: modA, modB,* and *modD*^27^,^28^. These share a similar overall structure, with N-terminal simple DNA repeats, N- and C-terminal domains that mediate DNA methylation, and a central DNA recognition domain (DRD) that determines the recognition and methylation site of the enzyme (See Fig. 1a). For each of the *modA, modB,* and *modD* genes, different alleles have been identified that have conserved N- and C-terminal domains (>90% amino acid identity), but which vary in the DRD sequence (>95% amino acid identity within alleles, and typically <40% identity among alleles). Different *mod* alleles (*i.e.* DRD variants) methylate different DNA sequences^28^,^30^,^32^ and regulate different phasevarions^27^,^28^. The *modA* gene has the highest known allelic diversity, with 20 known alleles (*modA1-20)*, many of which are found in *Haemophilus influenzae* (*modA1-10, modA14-17, modA20*) and/or *N. meningitidis* (*modA4, modA11-13, modA15, modA18-19*)^28^,^33^. In contrast, only four *modB* alleles have been reported: *modB1, modB2*^28^, *modB3*^34^ and *modB4*^15^. The *modB* gene has only been found in *Neisseria* species to-date, with *modB1* found in *N. meningitidis* and *N. gonorrhoeae, modB2* and *modB4* in *N. meningitidis*^28^, and *modB3* in *Neisseria lactamica*^29^. The *modD* gene also appears to be *Neisseria* specific, and has five known alleles: *modD1* and *modD2* in *N. meningitidis, modD3* in *N. lactamica, modD4* in *Neisseria cinerea* and *modD5* in *Neisseria mucosa*^27^. To date, the DNA methylation target sequences of *N. meningitidis* ModA11, ModA12 and ModD1^30^; *N. gonorrhoeae* ModA13 and ModB1^28^,^32^; *H. influenzae* ModA1, ModA2, ModA4, ModA5, ModA9 and ModA10^35^; and *M. catarrhalis* ModM2 and ModM3 have been determined^36^, with a unique site methylated by each allele. This study surveyed the distribution and combination of *mod* genes and their associated alleles in four *N. meningitidis* isolate collections, and identified their association with certain hyperinvasive lineages. These data are consistent with Mod proteins playing a role in the survival of meningococci in the different environments encountered during colonization and invasion in the human host.

## Results

### Distribution of *mod* genes and alleles

**Figure 1:**
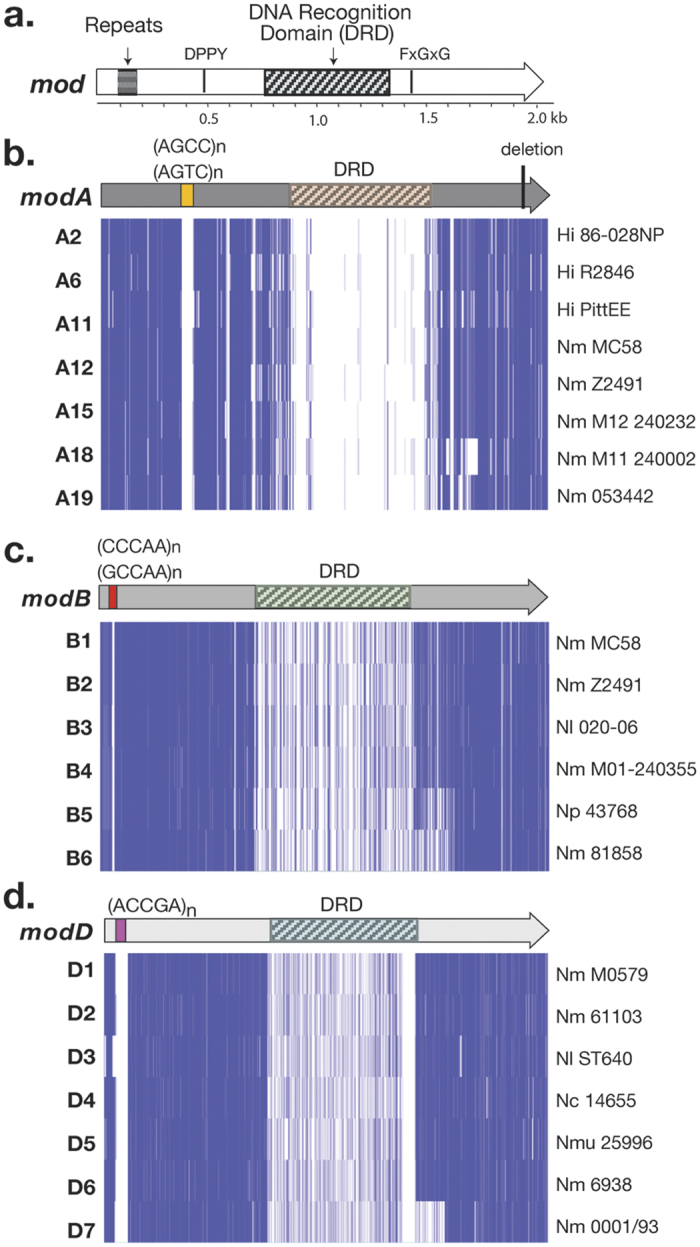
Key features of *mod* genes and alleles. (**a**) Schematic diagram of *mod* genes (represented by an arrow), showing the location of the phase variable DNA repeats, the DPPY and FxGxG motifs, and the central DNA recognition domain (DRD). For (**b**) *modA*, (**c**) *modB* and (**d**) *modD* genes, the location and type of phase variable repeats are shown, along with the location of the DRD and a graphical representation of a clustalW alignment of reference alleles [generated in JalView, with identical residues shown as vertical blue lines]. Strains that define the *mod* alleles are shown on the right (*Hi: Haemophilus influenzae; Nm: N. meningitidis; Nl: N. lactamica; Np: N. polysaccharea; Nc: N. cinerea; Nmu: N. mucosa*). For *modA* (**b**), the location of the 15-nucleotide deletion is also shown.

**Figure 2:**
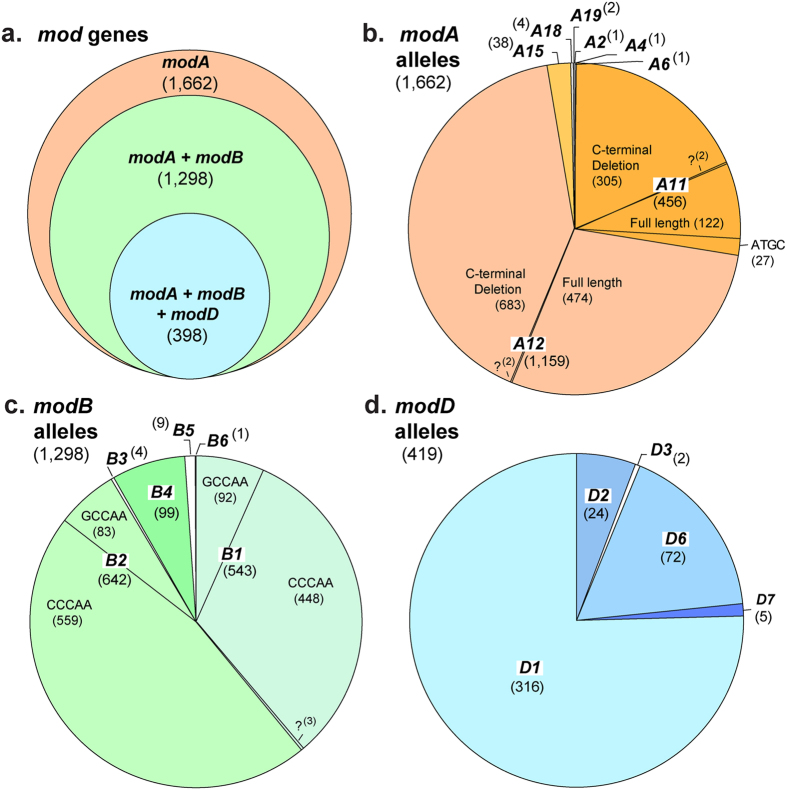
Distribution of *mod* genes and alleles. (**a**) Venn diagram of the distribution of *mod* genes in the dataset. (**b–d**) Pie charts of *mod* alleles (and allele variations) with the number of isolates containing each allele shown in brackets. (**b**) *modA* alleles 2, 4, 6, 11, 12, 15, 18, 19 are shown, as well as the A11 5′-AGTC-3 repeat variant, and the A11 and A12 15bp C-terminal deletion variants (NB. For 2 *modA11* and 2 *modA12* alleles it was unclear from whole genome sequences whether they were full length or had the C-terminal deletion, and these are indicated with a question mark ‘?’). (**c**) *modB* alleles 1–6 are shown, as well as the B1 and B2 5´-CCCAA-3´ and 5´-GCCAA-3´ repeat variants (NB. The repeat region could not be categorized for 3 *modB1* alleles, and these are indicated with a question mark ‘?’). (**d**) *modD* alleles 1–7 are shown.

To investigate the distribution of *mod* genes and alleles in *N. meningitidis* (Fig. 1), 1,689 isolates were surveyed, comprising 211 carriage and 1,478 disease isolates from four collections originating in diverse geographic locations (including the USA, UK, Czech Republic, and Australia) and time periods (1993–2013). These analyses determined that a *modA* gene was present in all isolates examined (although two isolates possessed only fragments of *modA*). A *modB* gene was identified in 78% (1,298) of isolates, while a *modD* gene was present in only 25% (423) of isolates (Fig. 2). Overall, 364 isolates contained *modA* only, 900 isolates contained both *modA* and *modB*, and 398 isolates contained *modA, modB* and *modD* genes. The number of DNA repeat units among isolates ranged from 2–34 tetranucleotide repeats in *modA*; 2–28 pentanucleotide repeats in *modB;* and 2–15 pentanucleotide repeats in *modD* (Fig. 1). Phase variation of tetranucleotide repeat tracts containing ≥3 repeat units occurs at a high frequency^37^, and the majority of these *mod* genes are predicted to be phase variable (>98% of fully assembled alleles contain ≥3 repeat units).

For each of the three *mod* genes, common and rare *mod* alleles (DRD variants) were found (Figs 1 and 2). The majority of *modA* positive isolates contained *modA12* (1,159 isolates, 70%) or *modA11* (456 isolates, 27.5%), with *modA15* comprising 2% of *modA* positive isolates (38 isolates) (Figs 1b and 2b). All other *modA* alleles were found at low frequency in the dataset, two of which, *modA2* and *modA6*, had not been reported in *N. meningitidis* before. In addition, several minor variations in the conserved regions of the *modA11* and *modA12* were seen among the isolates. The most frequent of these was a 15-nucleotide deletion (encoding [S(A/V)KNQ]) in the region encoding the C-terminus of ModA (Fig. 1b), found in 67% of *modA11* alleles and 59% of *modA12* alleles (Fig. 2b). This deletion removed 5 amino acids from the full length of the protein. In addition, the N-terminal, phase variable DNA repeat sequences were altered in some isolates. The typical *modA* repeat unit in *N. meningitidis* was 5′-AGCC-3′, however *modA11* in 27 isolates (6% of *modA11* isolates; 1.6% of total *modA* positive isolates) had 5′-AGTC-3′ repeats (Fig. 2b).

For *modB,* the most common alleles were *modB1* (543 isolates, representing 42% of all *modB* positive isolates) and *modB2* (642 isolates, 49%), with *modB4* present in 7.6% of *modB* positive isolates (Figs 1c and 2c). All other *modB* alleles were found at low frequency, including two new alleles (*modB5* and *modB6)*, which have not previously been defined. The *modB5* and *modB6* DRDs shared 11–48% identity at the deduced amino acid level with other *modB* alleles (Fig. 1c). Both were present at low frequency, with 9 *modB5* isolates (0.7%) and 1 *modB6* isolate (0.08%) identified. For *modB1* and *modB2,* the typical repeat unit was 5′-CCCAA-3′, with an alternate 5′-GCCAA-3′ repeat tract seen in 17% of *modB1* alleles and 13% of all *modB2* (Fig. 2c). *modB3* had both 5′-CCCAA-3′ and 5′-TCCAA-3′ repeats, and *modB4* and *modB5* typically contained 5′-GCCAA-3′ repeats.

The majority of *modD* positive isolates contained the *modD1* allele (316 isolates, 75%), or *modD6* (72 isolates, 17%; Figs 1d and 2d). All other *modD* alleles were found at low frequency, including *modD3* that has not been reported in *N. meningitidis* before, *modD6* that was previously identified in *N. meningitidis* 6938 but mis-categorized as *modD2*^38^,^39^, and *modD7* that has not previously been identified. The novel *modD7* allele was present in 5 isolates (1.2% of *modD* positive isolates), and shared 7–15% identity with other *modD* alleles over the length of the DRD (Fig. 1d). In addition, this allele had an extended DRD, with an additional 47 amino acids at the C-terminal end of the DRD, compared to other *modD* alleles (Fig. 1d).

Associations among different *mod* alleles were also identified. For example, of those meningococci containing only *modA* and *modB*, 71% of isolates with *modB1* also contained *modA11*, while 96% of isolates with *modB2,* and 89% of isolates with other *modB* alleles, were associated with *modA12.* Furthermore, 86% of isolates with a *modD* gene also contained *modA12* and *modB2*, which rose to 97% in isolates containing the *modD1* allele.

### Specific *mod* genes are associated with distinct clonal complexes

Examination of the distribution of *mod* in the dataset demonstrated that most *mod* genes and alleles were associated with specific sequence types and clonal complexes (Table 1). *ModA11, modA12, modB1* and *modB2* alleles were found in multiple clonal complexes and the majority of isolates from the same clonal complex contained the same allele. For example, *modA11* was present in cc32 (97% of cc32 isolates) and cc116 (85%), whereas *modA12* was present in cc11 (99%) and cc23 (97%) isolates. The C-terminal deletion variations were also clonal complex associated, with the *modA11* C-terminal deletion found in cc35 (82%), cc53 (91%) and cc269 (85%), and the *modA12* C-terminal deletion found in cc41/44 (97%) and cc213 (89%) meningococci. The *modB1* allele was found in cc269 (86%), and cc32 (100%), and the *modB2* allele was associated with cc41/44 (76%) and cc11 (100%) isolates; however, *modB* was absent in all cc23, cc53, cc92, cc106, cc116 and cc461 meningococci examined. *ModB1* and *modB2* sequences with non-typical repeat tracts were frequently found in cc60 (82%) and cc22 (81%), respectively. On the other hand, the less common alleles were associated with a single clonal complex, for example, *modA15* was associated with cc92 (present in 95% of cc92 meningococci vs. 0.1% of other clonal complexes, p < 0.0001), *modB4* with cc213 (90% vs. 0.3%, p < 0.0001), and *modD1* with cc41/44 (80% vs. 0%, p < 0.0001) (Table 1).

**Figure 3:**
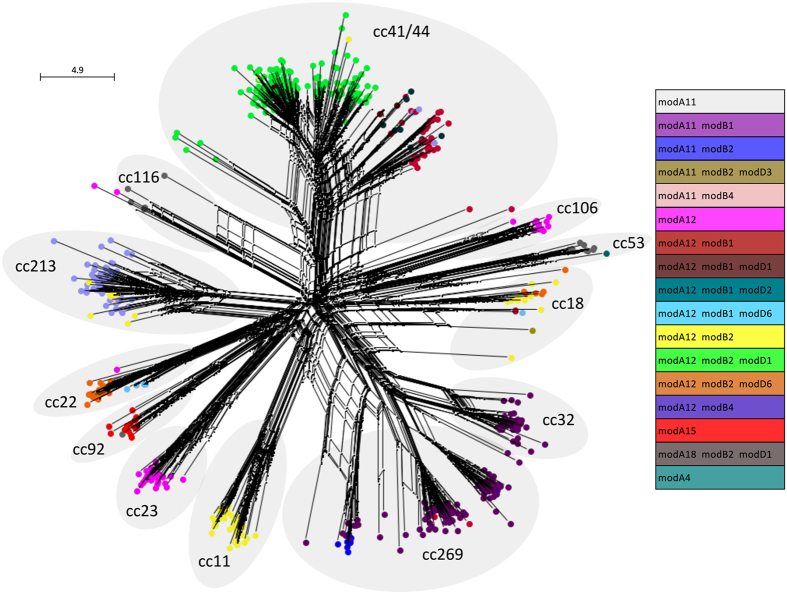
Association of *mod* alleles and clonal complexes. Neighbor-Net graph drawn from Ribosomal Multilocus Sequence Typing (rMLST) allele distances among unique Ribosomal Sequence Types (rSTs; n = 639) in clonal complexes comprising >10 isolates in the dataset. Isolates are colored based on the *mod* allele or allele combination present.

Further associations between *mod* alleles and clonal complexes were identified when *mod* allelic combinations were mapped onto a Ribosomal Multilocus Sequence Typing (rMLST) network of the twelve most frequently represented clonal complexes in this dataset (cc106, cc11, cc116, cc18, cc213, cc22, cc23, cc269, cc32, cc41/44, cc53 and cc92) with certain combinations more evident than others (Fig. 3, Supplementary Table 1). For example, *modA11* alone (*i.e.,* without *modB* or *modD*) was present in 91% of cc53 and 85% of cc116 isolates (vs. 1.8% of isolates from other complexes; p < 0.0001), *modA12* alone was present in 100% of cc106, 99% of cc23 and 100% of cc461 isolates (vs. 1.7% in other complexes, p < 0.0001), while *modA15* alone was present in 95% of cc92 isolates (vs. 0.1% in other complexes; p < 0.0001). The *modA11-modB1* combination was found in 89% of cc269 and 99% of cc32 isolates (vs. 1.2% in other complexes, p < 0.0001); while the *modA12*-*modB2* combination was found in 99% of cc11 isolates (vs. 5.2% in other complexes, p < 0.0001); however, the *mod-* clonal complex associations were not always completely conserved. For example, cc18 isolates did not share a common allelic combination, and cc41/44 isolates were clustered into two groups, one including ST-41 and the other ST-44 isolates. The ST-41 cluster was associated with *modA12, modB2* and *modD1* (78% of isolates, with this combination not seen in other complexes, p < 0.0001) whereas the ST-44 cluster was associated with *modA12* and *modB2* but lacked *modD1* (16% of ST-44 isolates vs. 3.8% in other complexes, p < 0.0001; Fig. 3). Similarly, most cc22 isolates contained the *modA12-modB2-modD6* combination, but a smaller cluster of isolates had *modA12-modB1-modD6*. The majority of cc269 isolates possessed *modA11-modB1*, however one cluster was associated with *modA11-modB2* (Fig. 3).

### Specific *mod* genes and combinations are associated with invasive or carriage meningococci

Given that different *mod* alleles regulate distinct sets of genes (phasevarions) that affect the phenotype of the isolate, the distribution of *mod* alleles relative to the isolate’s disease outcome (*i.e.,* invasive disease or carriage) was considered. Several associations were observed among *mod* alleles and invasive or carried meningococci, and these associations were particularly strong for atypical alleles and allelic combinations. For example, *modA11* and *modB2* were more commonly associated with invasive, rather than carriage, isolates (respectively, 29% vs. 13% for *modA11* (p < 0.0001); 40% vs. 28% for *modB2* (p = 0.002); Table 2). The *modA12-modB2-modD1* combination was associated with cc41/44 invasive isolates (20% invasive vs. 0% carriage, p < 0.0001), while carriage isolates from this clonal complex possessed *modA12-modB1* but lacked *modD1* (87% carriage vs. 9% invasive with *modA12-modB1*, p < 0.0001) (Fig. 3). The *modA12-modB4* combination was associated with cc213 invasive isolates and was only found in two carriage isolates of different sequence types (6.5% of invasive isolates vs. 1% carriage, p = 0.0004). Also, the *modA11*-*modB1* combination was associated with invasive isolates, and present in hyperinvasive lineages cc32 and cc269 (22% of invasive vs. 2.4% carriage isolates, p < 0.0001). The atypical *modA15* allele was usually found alone and in cc92 carriage isolates (12% of total carriage isolates vs. 0.2% of invasive isolates, p < 0.0001).

## Discussion

The phase variable type III DNA methyltransferases encoded by the *modA, modB* and *modD* genes are a global control mechanism by which *N. meningitidis* can randomly alter the expression of distinct sets of genes, known as phasevarions^29^. Several alleles of each of the *mod* genes have been characterized, based on differences in the region encoding the DNA recognition domain (DRD), each of which methylates a different sequence and mediates the epigenetic regulation of different sets of genes^28^,^30^. Many isolates contain multiple *mod* genes (*i.e.,* have multiple phasevarions) which can phase vary independently. This enables the reversible induction of polygenetic phenotypes, which increases bacterial adaptability to distinct ecological environments and may affect invasive capacity. For example, the phase variable ON/OFF switching of meningococcal ModD1 alters resistance to oxidative stress^27^, while phase variation of ModA11 and ModA12 results in altered antibiotic resistance^40^. Similarly, previous studies of the gonococcal ModA13 phasevarion have shown that ON/OFF variants have distinct phenotypes for biofilm formation, resistance to antimicrobials, and survival in primary human cervical epithelial cells^28^.

Our analysis of the distribution of *mod* genes and alleles in meningococci isolated from carriage and invasive disease revealed high levels of diversity in type III DNA methyltransferases and their respective DRD regions, and identified associations between *mod* alleles and clonal complexes. These clonal complexes included both hyperinvasive lineages, responsible for the majority of disease worldwide (*e.g.,* cc11, cc32, cc41/44, and cc269), as well as those that are rarely associated with invasive disease (*e.g.,* cc35, cc92, cc106 and cc116)^7^,^8^,^9^,^10^,^41^. While some alleles, such as *modA12,* were found in both hyperinvasive (cc11 and cc41/44) and non-invasive (cc106) lineages, some alleles were more commonly associated with one or the other. For example, in terms of hyperinvasive lineages, *modA11* was associated with cc32 isolates, *modB1* with cc269 and cc32 isolates, and *modD1* with cc41/44 isolates. This is consistent with previous studies conducted in smaller datasets^27^,^28^. The *modA11*-*modB1* combination was associated with hyperinvasive lineages cc32 and cc269; and the *modA12-modB2-modD1* combination with the hyperinvasive ST-41 cluster. On the other hand, the *modA15* allele was predominantly found in isolates of the cc92 lineage, which is a non-invasive lineage. Furthermore, the distribution of *mod* alleles relative to the disease outcome or phenotype of each isolate (*i.e.,* invasive or carriage) showed associations of atypical alleles and allelic combinations with invasive or non-invasive meningococci. For example, *modA11*-*modB1, modA12-modB2-modD1* and *modA12-modB4* combinations were associated with invasive isolates, while *modA15* or the *modA12-modB1* combination were associated with isolates from carriage collections. While the *mod* alleles could be considered a marker of clonal complex rather than infection outcome, it is important to note that there is not always a strict correlation between clonal complex and infection outcome. This is highlighted within the cc41/44 lineage that contains two central STs, where ST-41 meningococci are commonly associated with invasive disease, while ST-44 are typically associated with carriage^10^. The distribution of *modD1* in this clonal complex is specifically associated with invasive disease; *modD1* is present in 84% of cc41/44 invasive isolates (105/105 ST-41 and 203/259 other STs) compared to 0% of cc41/44 carriage isolates (0/8 ST-44 and 0/22 other STs). Future experimental work will clarify whether the observed associations directly correspond to a difference in the phenotype and virulence of strains. An increased focus should also be placed on collecting and sequencing carriage isolates, as to date these are underrepresented in the available meningococcal isolate panels.

Several questions remain regarding the evolution of *mod* diversity, the frequency of *mod* allele mobilization, and whether the repertoire is being neutrally inherited, or selectively maintained. These data suggest that the more commonly found alleles (*e.g., modA11, modA12, modB1* and *modB2*), along with their allelic combinations, may have been acquired early in the evolution of *N. meningitidis*, and have been successively inherited by vertical transmission from progenitor cells that have differentiated into multiple clonal complexes. The rarer alleles, and differences in *mod* allele combinations, may have arisen from more recent horizontal gene transfer (HGT) and recombination events common to *Neisseria*^13^,^38^,^39^,^41^,^42^,^43^. For example, the discovery of single isolates containing the *modA2, modA4, modA6,* and two isolates with *modD3* alleles is consistent with HGT in *N. meningitidis*. Transfer and replacement of alleles is possibly enhanced by the structure of the *mod* genes, as the conserved flanking regions may facilitate homologous recombination and replacement of the central variable DRD. This has previously been reported for the *modA* gene, with sequence analysis suggesting some DRDs originated from other bacterial species^33^. Similarly, the distribution of several alleles (*e.g., modA2, modA6, modD3* identified in *N. meningitidis* for the first time) suggests horizontal mobilization of DNA from outside the species^44^,^45^,^46^, as these alleles have been previously identified in *H. influenzae, N. polysaccharea* and *N. lactamica*^27^,^29^,^33^. In order to investigate the temporal and geographical nature of the associations seen, more diverse and long-term isolate collections are needed. However, it can be noted that the some associations, such as that seen between cc11 isolates and *modA12* and *modB1,* are consistent in all the collections over the time period, while others are more specific to certain locations but may reflect the invasive or carriage focus of the collections, for example, the cc92 carriage isolates associated with the *modA15* allele are largely isolates from 1993 from the Czech carriage study: whereas *modD1* is associated with invasive isolates from the UK and Australian collections.

The evolution and biological significance of the 15-nucleotide C-terminal deletion and the DNA repeat tract variants are unknown. The C-terminal deletion does not affect the well-described conserved motifs of type III Mods, such as the catalytic region (DPPY) and the S-adenosyl-L-methionine methyl donor-binding pocket (FXGXG) (See Fig. 1a)^29^,^47^,^48^, and ModA12 has been shown by methylome analysis to be functional in strains with (M0579) or without (B6116/77) this deletion^30^. It is noteworthy, however, that the typical 5′-AGCC-3′ *modA* repeat tract is recognized by the ModD methyltransferase DNA recognition domain. Previous studies propose that methylation within gene coding regions may alter transcription^45^: this may suggest that variations arise as a mechanism to circumvent methylation of the repeat tract.

Previous studies posit that meningococcal restriction-modification systems, such as the type III systems that the *mod* genes are part of, are clade associated, and maintain barriers to gene transfer between groups^15^; however, other studies suggest that genome recombination is more frequent than expected^38^, and that restriction-modification systems do not necessarily prevent genetic transfer, either because they are being mobilized themselves, or due to transient or inefficient function^39^. This latter point may be particularly true for the *mod* alleles, given that Mod is phase variable and the restriction enzyme is dependent on the presence of Mod in type III restriction-modification systems, and that inactivating mutations can be found in the cognate restriction enzymes in some systems^28^,^49^. If this is indeed the case, then the selective maintenance of the *mod* allele repertoire may be attributable to the epigenetic regulatory functions of Mod, as has previously been suggested to explain the dominance of the *modA12* allele in *N. meningitidis*^33^. To support this hypothesis, full characterization of strains, and the phasevarions regulated by Mod proteins is needed. These studies will clarify the significance of *mod* allele and clonal complex associations, and how ON/OFF switching affects the phenotype of *N. meningitidis*, especially in light of the fact that isolates can contain up to three distinct *mod* alleles and phasevarions. Given that each *mod* allele can phase vary independently, each isolate can give rise to eight possible distinct sub-populations. These variants would appear genetically identical, differing only in the loss or gain of a few repeat units in the *mod* gene, but would have profound differences in gene expression and potentially in their invasive phenotype. It is also important to note that the potential fluidity of allelic exchange of *mod* alleles means that with a single gene recombination event, *N. meningitidis* strains may acquire a new *mod* allele and the ability to regulate genes in a different phasevarion, and consequently display a different phenotypic profile.

In terms of *mod* allele associations with invasive meningococci, it is important to note that disease does not increase the fitness of meningococci as there is no corresponding benefit to transmission^50^. However, if certain Mod proteins do increase the invasiveness of certain clonal complexes, the fact that Mod expression is phase variable would allow the Mod regulatory system to be maintained. For example, even under conditions where Mod ON is deleterious or increases the probability of invasion, cells with Mod in phase OFF may persist, in which case, selective pressure against *mod* is removed without affecting the future function of the Mod regulatory system or bacterial fitness. Accordingly, these alleles provide a mechanism that enables cells to variably express complex phenotypes, which may facilitate a transition from carriage to invasion and *vice versa* depending on environmental changes. If so, this may help elucidate how transmissibility and virulence are linked in meningococcal lineages^43^. Hence, the study of these regulators, and how they change over time, may provide critical insights into how and why *N. meningitidis* cells generate distinct phenotypes without overt changes in gene content, and how this may mediate transition from carriage to invasive disease. While it is tempting to speculate on the ON vs. OFF status of the Mods from the available genome sequences, it is important to note that the samples used for sequencing were not collected for this purpose and are therefore not an accurate reflection of the natural ON/OFF status and ratio of the *in vivo* bacterial population. Therefore, unlike past epidemiological studies that typically isolate and characterize single meningococcal colonies from patient samples, future studies will require direct and unbiased sequencing, or the isolation of representative populations, of bacteria from blood, cerebrospinal fluid and the nasopharynx. The characterization of the presence and expression state of individual *mod* alleles in these samples will help determine the significance of *mod* allele distribution relative to meningococcal carriage and disease.

## Methods

### *N. meningitidis* isolate collections

Four *N. meningitidis* isolate collections were analyzed in this study: i) 20 previously characterized whole-genome sequences (WGS) from 18 disease and two carriage isolates^12^,^15^,^25^,^51^,^52^,^53^; ii) WGS from 54 disease and 209 carriage isolates from the Czech Republic^41^,^43^; iii) WGS data from 1380 disease isolates in the Meningitis Research Foundation (MRF) Meningococcus Genome Library (MGL) (www.meningitis.org/current-projects/genome)^62^; and, iv) 50 disease isolates from Australia^54^.

### Screening for *mod* genes

The *mod* genes were identified by bioinformatic analysis of WGS data using the BIGSdb platform hosted on pubmlst.org/neisseria^55^. Locus records for *modA* (NEIS1310), *modB* (NEIS1194) and *modD* (NEIS2364) were generated in the PubMLST database (http://pubmlst.org/neisseria)^55^ using previously identified reference *modA* and *modB* nucleotide sequences from *N. meningitidis* MC58^28^,^53^ and the *modD* sequence from *N. meningitidis* M0579^15^,^27^. The BIGSdb ‘Scan Tag’ tool^56^ was implemented for the identification of *mod* loci within WGS data of each isolate, returning BLAST matches greater than 30% alignment and 50% identity to the sequences stored in locus records. The nucleotide diversity of *mod* loci required that hits be exported to MEGA6^57^ for manual alignment with previously identified (see reference alleles below) full length *mod* coding sequences and DNA recognition domain (DRD) based alleles. For example, alignment gaps were inserted in the phase variable repeat region of phase OFF sequences to allow comparison with the full-length translated amino acid sequence. Trimmed sequences were uploaded to the appropriate locus record for storage where they were assigned unique numeric identifiers (allele id numbers), and grouped into variants based on the DRD (corresponding to the *mod* alleles described below). Database alleles were flagged with information such as phase-variation status and where the open reading frame was interrupted due to insertions, deletions or point mutations (other than changes to the number of phase variable repeat units), and database alleles were assigned the flags ‘internal stop codon’ and ‘frameshift’ where necessary. These interrupted alleles were not included in the analysis of invasive versus carriage meningococci. The BIGSdb ‘autotagger’ and ‘autodefiner’ tools^56^ were then used to identify *mod* genes within genomic data stored for each isolate, allowing tagging of nucleotide positions and database allele id numbers.

The *mod* loci were frequently present on different contiguous sequences of a genome assembly due to break points within phase variable regions or insertion sequences: this permitted identification of the presence of a *mod* gene and its allele, but not assignment of a database allele id number. In these cases, a database flag was inserted at these genomic positions to indicate a partial assembly, and isolates were considered to contain *mod* genes but were not included in subsequent allele analyses. This process was performed iteratively until no new alleles of the three *mod* genes could be identified in the genomes; at this stage, genomes without tagged *mod* genes were investigated using implementations of the BIGSdb BLAST tool (parameters: word size 11; reward 2; penalty -3; gap open 5; gap extend 2). For each locus variant, six hits were investigated per isolate, regardless of E-value significance, and 1000 bp of flanking sequence were extracted with the hit for investigation in MEGA6; new alleles were uploaded to the database as before, otherwise genomes were tagged as ‘missing’ *mod* loci.

To screen for *mod* genes and alleles in the Australian disease isolates (no WGS available), PCR and sequencing analyses was performed as previously described^27^,^28^. MLST analysis was performed in accordance with the scheme guidelines (http://pubmlst.org/neisseria/info/)^55^.

Sequence alignments were performed using ClustalW^58^ and were exported into Jalview^59^ to generate alignment Figures. The Neighbor-Net graph was generated in SplitsTree4^60^ from allelic distances among the 49 rMLST loci of unique ribosomal Sequence Types (rSTs) (n = 639)^61^ among clonal complexes comprising >10 isolates in the dataset. rSTs (available at http://pubmlst.org) were annotated with the combination of *mod* alleles present in isolates, and clusters were labeled according to the clonal complexes of these isolates. 186 isolates were not assigned to a known clonal complex.

Statistical analyses were carried out using 2-tailed Fisher’s Exact tests (Graphpad Software Inc., San Diego, CA, USA), with p-values of p < 0.05 taken to indicate statistical significance.

### *mod* gene/allele reference sequences

The *modA, modB*, or *modD* genes were defined by >90% sequence identity along the length of the deduced amino acid sequence, excluding the variable DRD, to the *modA11* and *modB1* genes of *N. meningitidis* MC58^28^, or the *modD1* gene of M0579^27^. The alleles of each *mod* gene were defined by ≥95% amino acid identity across the DRD to the reference sequences listed below.

For *modA,* reference sequences were as previously described^28^,^33^: *modA2, H. influenzae (Hi)* strain 723*; modA4, Hi* 3579*; modA6, Hi* C1626; *modA11, N. meningitidis* (*Nm*) MC58; *modA12, Nm* Z2491; *modA15, Hi* R3570; *modA18, Nm* NGE28; *modA19, Nm* 053422. For *modA* allele alignments, sequences from the following isolates were used for the full-length gene reference: *modA2, Hi* 86-028NP*; modA4, Hi* R2846*; modA6, Hi* PittEE. For *modA15* and *modA18*, no full-length genes were available in GenBank, and isolates M12 240232 and M11 240002 from the MRF MGL have been used, respectively.

For *modB,* reference sequences were as previously described^29^: *modB1, Nm* MC58; *modB2, Nm* Z2491; *modB3, N. lactamica* (*Nl*) 020-60. For *modB4, Nm* M01-240355^15^. For newly described *modB5* and *modB6*, GenBank database matches were identified in *N. polysaccharea* 43768 (NEIPOLOT_01008) for *modB5*; and *Nm* 81858 (NM81858_1294) for *modB6.*

For *modD*, reference sequences were as previously described^27^: *modD1, Nm* M0579; *modD3, Nl* ST640; *modD4, N. cinerea* 14655; *modD5, N. mucosa* 25996. For *modD2, Nm* 61103 (NM61103_0875). For *modD6, Nm* 6938^38^. For *modD7*, the Czech isolate *Nm* 0001/93^43^ was used from the MRF MGL as no similar sequences were identified in GenBank.

## Additional Information

**How to cite this article**: Tan, A. *et al.* Distribution of the type III DNA methyltransferases *modA, modB* and *modD* among *Neisseria meningitidis* genotypes: implications for gene regulation and virulence. *Sci. Rep.* **6**, 21015; doi: 10.1038/srep21015 (2016).

## Supplementary Material

Supplementary Information

## Figures and Tables

**Table 1:** Associations between *mod* alleles and clonal complexes.

Gene (Locus recordnumber)^a^ Allele^b^	Predominant clonal complex associated with allele			
		cc^c^	% (#)^d^	p-value^f^
*modA* (NEIS1310)				
* modA11*	Full length	cc32	97 (72/74)	<0.0001
		cc116	85 (11/13)	<0.0001
	C-terminal deletion^e^	cc35	82 (14/17)	<0.0001
		cc53	91 (10/11)	<0.0001
		cc269	85 (231/272)	<0.0001
	5′-AGTC-3′ repeats^e^	cc162	100 (23/23)	<0.0001
* modA12*	Full length	cc11	99 (175/176)	<0.0001
		cc23	97 (173/179)	<0.0001
	C-terminal deletion^e^	cc41/44	97 (363/368)	<0.0001
		cc213	89 (90/101)	<0.0001
* modA15*		cc92	95 (21/22)	<0.0001
*modB* (NEIS1194)				
* modB1*	5´-CCCAA-3´ repeats	cc32	100 (74/74)	<0.0001
		cc41/44	16 (60/368)	<0.0001
		cc269	86 (233/272)	<0.0001
	5′-GCCAA-3′ repeats^e^	cc60	82 (18/22)	<0.0001
		cc269	8 (22/272)	0.0016
* modB2*	5´-CCCAA-3´ repeats	cc11	100 (176/176)	<0.0001
		cc41/44	76 (281/363)	<0.0001
	5′GCCAA-3′ repeats^e^	cc22	81 (35/43)	<0.0001
		cc174	93 (14/15)	<0.0001
* modB4*		cc213	90 (91/101)	<0.0001
* modB5*		cc282	100 (9/9)	<0.0001
* *No *modB*		cc23	100 (179/179)	<0.0001
		cc461	100 (31/31)	<0.0001
*modD* (NEIS2364)				
* modD1*		cc41/44	80 (308/384)	<0.0001
* modD2*		cc41/44	2 (9/384)	0.0001
* modD6*		cc18	30 (7/23)	<0.0001
		cc22	98 (44/45)	< 0.0001

^a^*mod* genes and corresponding locus record NEIS numbers in the PubMLST database (http://pubmlst.org/neisseria/).

^b^Alleles were determined based on >95% sequence identity to representative type sequences outlined in the material and methods.

^c^Predominant clonal complexes containing the respective *mod* allele, with complexes, determined by the *Nm* MLST scheme (http://pubmlst.org/neisseria/). Note that this analysis was restricted to isolates with full allele, and cc data available.

^d^% of isolates from the clonal complex with the allele (number of isolates with the allele/total number of isolates from the clonal complex).

^e^Variations to the reference allele. C-terminal deletion: missing 15 nucleotides near the C-terminal of the gene. 5′-AGTC-3′ and 5′GCCAA-3′ repeats: contain different repeat tract sequences to the reference allele. Further details on allele variations are given in the text.

^f^p-value for the association of the given allele with the clonal complex listed (vs. other clonal complexes), calculated using the Fisher’s Exact test (two-tailed).

**Table 2:** Distribution of individual alleles and allele combinations in carriage and invasive meningococci.

Allele			Isolates with allele^a^	% Carriage (#)^b^	cc(s)^c^	% Invasive (#)^b^	cc(s)^c^	p-value^d^
*modA11*			454	13 (28)	18, 32, 53, 116, 269	29 (426)	23, 32, 41/44, 92, 213, 269	<0.0001
*modA12*			1,135	70 (148)	11, 18, 22, 41/44. 106, 116	67 (987)	11, 18, 22, 23, 41/44, 213, 269	0.3478
*modA15*			35	12 (25)	92	1 (10)		<0.0001
*modB1*			465	23 (49)	18, 32, 41/44, 269	28 (416)	18, 22, 32, 41/44, 269	0.1389
*modB2*			634	28 (60)	11, 18, 22	40 (574)	11, 18, 22, 41/44, 213, 269	0.0018
*modB4*			97	1 (2)	41/44	6.5 (95)	41/44, 213	0.0004
*modD1*			315	0 (0)		21 (315)	41/44	<0.0001
*modD6*			71	8 (17)	18, 22	3 (54)	18, 22, 41/44	0.0014
**Allele combination**			**Isolates with combo**^a^	**% Carriage (#)**^b^	**cc(s)**^c^	**% Invasive (#)**^b^	**cc(s)**^c^	**p-value**^d^
***modA***	***modB***	***modD***						
*modA11*	–	–	109	9.5 (20)	53, 116	6 (89)	23, 32, 92, 269	0.1743
*modA12*	–	–	282	19 (40)	22, 106, 116	17 (242)	22, 23, 106, 116, 213,	0.4326
*modA15*	–	–	28	12 (25)	92	0.2 (3)		<0.0001
*modA11*	*modB1*	–	317	2.4 (4)	32, 269	22 (313)	32, 269	<0.0001
*modA12*	*modB1*	–	103	15 (31)	41/44	5 (72)	18, 41/44, 269	<0.0001
*modA12*	*modB2*	–	257	22 (47)	11, 18	15 (210)	11, 18, 41/44, 213	0.057
*modA12*	*modB4*	–	94	1 (2)	41/44	6.5 (92)	41/44, 213	0.0004
*modA12*	*modB2*	*modD1*	286	0 (0)		20 (286)	41/44	<0.0001
*modA12*	*modB2*	*modD6*	45	3 (7)	22	3 (38)	18, 22, 41/44	0.6514

^a^Total number in the dataset with each of the most frequent *modA, modB* and *modD* alleles, or allele combinations. (−) indicates the *mod* gene is absent.

^b^% (total number) of carriage or invasive isolates with the *mod* allele or allele combination, calculated as a proportion of total carriage or invasive isolates, respectively. For these analyses, alleles/isolates were included if full allele data was available, and if the allele was considered to encode a potentially functional methyltransferases (*i.e.,* contained no point mutations or insertions/deletions (other than insertions/deletions in the phase variable repeat region); *e.g.,* an allele profile of *modA11 - modB1::IS1301* was considered to only contain a putatively functional *modA11*). Total number of isolates included in analyses: 211 carriage and 1478 invasive isolates for allele distribution; 211 carriage and 1448 invasive isolates for allele combinations (30 invasive isolates removed due to incomplete allele combination data).

^c^Predominant clonal complexes containing the allele or allele combination. Clonal complexes are only included if they contain >10 isolates in the database. Sequence types not assigned to a clonal complex are excluded.

^d^p-value for the association of the given the allele or allele combination with either carriage or invasive isolates, calculated using the Fisher’s Exact test (two-tailed).
