# Supplementary material for: Distribution of the type III DNA methyltransferases modA, modB and modD among Neisseria meningitidis genotypes: implications for gene regulation and virulence
Source: Sci Rep. 2016 Feb 12;6:21015. doi: 10.1038/srep21015 (PMC4751487; doi:10.1038/srep21015)
Supplement: Supplementary Information [file srep21015-s1.pdf]

Supplementary data for:

Distribution of the type III DNA methyltransferases *modA, modB* and *modD* among *Neisseria meningitidis* genotypes: implications for gene regulation and virulence

Aimee Tan[1#], Dorothea M. C. Hill[2#], Odile B. Harrison[2], Yogitha N. Srikhanta[1^], Michael P. Jennings[1], Martin C. J. Maiden[2], Kate L. Seib[1*]

[1]Institute for Glycomics, Griffith University, Gold Coast, Queensland 4222, Australia.

[2]Department of Zoology, University of Oxford, Oxford, UK.

[#] Contributed equally to this work

^ Current Address: Faculty of Medicine, Nursing and Health Sciences, Monash University, Victoria, 3800, Australia.

* Corresponding author: Institute for Glycomics, Griffith University, QLD, Australia. E-mail: k.seib@griffith.edu.au

Supplementary Table 1. Allele combinations and their commonly associated clonal complexes (cc)

Allele			isolates with combo[a]	Common cc	Combo frequency within cc % (#)[b]	Combo frequency outside cc % (#)[c]	p-value[d]
modA	*modB*	*modD*					
modA11	-	-	47	cc116	85 (11/13)	1.8 (26/1455)	<0.0001
				cc53	91 (10/11)		
modA11	*modB1*	-	330	cc269	89 (243/272)	1.2 (14/1133)	<0.0001
				cc32	99 (73/74)		
modA12	-	-	248	cc23	99 (178/179)	1.7 (21/1251)	<0.0001
				cc106	100 (18/18)		
				cc461	100 (31/31)		
modA12	*modB1*	-	99	cc41/44	16 (57/366)	3.8 (42/1113)	<0.0001
modA12	*modB2*	-	251	cc11	99 (175/176)	5.2 (66/1281)	<0.0001
				cc18	45 (10/22)		
modA12	*modB2*	*modD1*	285	cc41/44	78 (285/366)	0 (0/1113)	<0.0001
modA12	*modB2*	*modD6*	42	cc22	81 (35/43)	0.5 (7/1436)	<0.0001
modA12	*modB4*	-	92	cc213	89 (89/100)	0.2 (3/1379)	<0.0001
modA15	-	-	23	cc92	95 (21/22)	0.1 (2/1457)	<0.0001

[a] Number of isolates with allele combination (NB. Only isolates with assigned clonal complexes (1479 isolates of the 1689 in the database) are included).

[b] % of isolates from the clonal complex with the *mod* allele combination (number of isolates with the combination from the clonal complex / total number of isolates from the clonal complex).

[c] % of isolates from other clonal complexes with the *mod* allele combination (number of isolates in the listed clonal complexes with the combination / total number of isolates from other clonal complexes with the combination).

[d] p-value for the association of the given allele combination with the clonal complex listed (vs. other clonal complexes), calculated using the Fisher's Exact test (two-tailed).
